# Neuroprotective effects of aldehyde dehydrogenase 2 activation in rotenone-induced cellular and animal models of parkinsonism

**DOI:** 10.1016/j.expneurol.2014.09.016

**Published:** 2014-09-28

**Authors:** Ching-Chi Chiu, Tu-Hsueh Yeh, Szu-Chia Lai, Yah-Huei Wu-Chou, Che-Hong Chen, Daria Mochly-Rosen, Yin-Cheng Huang, Yu-Jie Chen, Chao-Lang Chen, Ya-Ming Chang, Hung-Li Wang, Chin-Song Lu

**Affiliations:** a Neuroscience Research Center, Chang Gung Memorial Hospital at Linkou Medical Center, Taoyuan, Taiwan; b Section of Movement Disorders, Department of Neurology, Chang Gung Memorial Hospital at Linkou Medical Center, Taoyuan, Taiwan; c Healthy Aging Research Center, College of Medicine, Chang Gung University, Taoyuan, Taiwan; d College of Medicine, Chang Gung University, Taoyuan, Taiwan; e Human Molecular Genetics Laboratory, Chang Gung Memorial Hospital at Linkou Medical Center, Taoyuan, Taiwan; f Department of Chemical and Systems Biology, Stanford University School of Medicine, Stanford, CA, USA; g Department of Neurosurgery, Chang Gung Memorial Hospital at Linkou Medical Center, Taoyuan, Taiwan; h Department of Physiology and Pharmacology, College of Medicine, Chang Gung University, Taoyuan, Taiwan

**Keywords:** Alda-1, aldehyde dehydrogenase 2 (ALDH2), mitochondrial dysfunction, Parkinson’s disease, rotenone, substantia nigra dopaminergic neurons

## Abstract

Many studies have shown that mitochondrial aldehyde dehydrogenase 2 (ALDH2) functions as a cellular protector against oxidative stress by detoxification of cytotoxic aldehydes. Within dopaminergic neurons, dopamine is metabolized by monoamine oxidase to yield 3,4-dihydroxyphenylacetaldehyde (DOPAL) then converts to a less toxic acid product by ALDH. The highly toxic and reactive DOPAL has been hypothesized to contribute to the selective neurodegeneration in Parkinson’s disease (PD). In this study, we investigated the neuroprotective mechanism and therapeutic effect of ALDH2 in rotenone models for parkinsonism. Overexpression of wild-type ALDH2 gene, but not the enzymatically deficient mutant ALDH2*2 (E504K), reduced rotenone-induced cell death. Application of a potent activator of ALDH2, Alda-1, was effective in protecting against rotenone-induced apoptotic cell death in both SH-SY5Y cells and primary cultured substantia nigra (SN) dopaminergic neurons. In addition, intraperitoneal administration of Alda-1 significantly reduced rotenone- or MPTP-induced death of SN tyrosine hydroxylase (TH)-positive dopaminergic neurons. The attenuation of rotenone-induced apoptosis by Alda-1 resulted from decreasing ROS accumulation, reversal of mitochondrial membrane potential depolarization, and inhibition of activation of proteins related to mitochondrial apoptotic pathway. The present study demonstrates that ALDH2 plays a crucial role in maintaining normal mitochondrial function to protect against neurotoxicity and that Alda-1 is effective in ameliorating mitochondrial dysfunction and inhibiting mitochondria-mediated apoptotic pathway. These results indicate that ALDH2 activation could be a neuroprotective therapy for PD.

## Introduction

Parkinson’s disease (PD) is the second most common neurodegenerative disorders, which affects 1 to 2% of the population above 65 years of age, and characterized by resting tremors, muscle rigidity, bradykinesia and postural abnormalities (Lees et al., 2009, Olanow et al., 2009). The neuropathological hallmarks of PD are the progressive degeneration of dopaminergic neurons in the substantia nigra pars compacta (SNpc) and the appearance of cytoplasmic inclusions termed as Lewy bodies. Although the molecular pathogenesis of PD is not completely understood, mitochondrial dysfunction, inflammation, excitotoxicity, elevated oxidative stress, aldehyde-related toxicity, and environmental toxins play important roles in the pathogenesis of PD (Thomas and Beal, 2007, Schapira, 2008, Martin et al., 2011, Tsuboi, 2012). Mitochondrial dysfunction and subsequent oxidative stress result in the generation of reactive oxygen species (ROS). These processes contribute to neurodegeneration via lipid peroxidation, which consequently lead to the production of reactive aldehydes, such as 3,4-dihydroxyphenylacetaldehyde (DOPAL), malondialdehyde (MDA), and 4-hydroxy-2-nonenal (4-HNE) (Ohta et al., 2004, Marchitti et al., 2007). DOPAL, which is an intermediate product of dopamine (DA) metabolism, has been suggested to contribute to the pathogenesis of PD (Marchitti et al., 2007, Panneton et al., 2010, Goldstein et al., 2013). 4-HNE is one of most important products of peroxidation of cellular membrane lipids, or circulating lipoprotein molecules during oxidative stress. The presence of 4-HNE is increased in brain tissue in neurodegenerative disorders such as Alzheimer’s disease (AD), Amyotrophic lateral sclerosis (ALS), and PD (Zarkovic, 2003). These reactive aldehydes are neurotoxic and form adducts with proteins, leading to neuronal death in PD.

Aldehyde dehydrogenases (ALDHs; EC1.2.1.3), consisting of 19 putatively functional genes with distinct chromosomal locations in human, represent a group of enzymes that are critical for certain life processes and detoxification via the NAD(P)^+^-dependent oxidation of a wide range of endogenous and exogenous aldehyde substrates to their corresponding carboxylic acids. Mitochondrial aldehyde dehydrogenase 2 (ALDH2) is highly expressed in heart and brain (tissues with a high mitochondrial content) and is found in the dopaminergic neurons of midbrain (McCaffery and Drager, 1994, Zimatkin and Karpuk Yu, 1996). Within dopaminergic neurons, ALDH2 is the principal enzyme involved in detoxifying aldehydes, including DOPAL, 4-HNE and other ROS-induced aldehydes by converting them to a less toxic acid products (Ohta et al., 2004, Marchitti et al., 2007, Chen et al., 2014, Doorn et al., 2014). The prevalent single point mutation of human ALDH2 gene found in a large population of the East Asian countries substitutes a glutamate with lysine at amino acid position 504 (E504K, formerly E487K that the 17-amino acid mitochondrial targeting sequence is not included). The E504K mutant allele, denoted as ALDH2*2, accounts for 35–57% of East Asians (Li et al., 2009). The ALDH2*2 mutation results in a drastic reduction in ALDH2 enzymatic activity to about 1–3% in homozygous ALDH2*2/*2 individuals and 17–38% in heterozygous ALDH2*1/*2 individuals as compared to the wild type individuals (denoted as ALDH2*1/*1) (Yoshida et al., 1984, Lai et al., 2014). Epidemiological studies suggest that ALDH2 E504K mutation increases the risk of Alzheimer’s disease among East Asian men (Hao et al., 2011). Transgenic mice over-expressing ALDH2*2 have shortened lifespan and exhibit age-dependent neurodegeneration and hyperphosphorylation of tau in hippocampal neurons (Ohsawa et al., 2008). In addition, *Aldh1a1*^−/−^×*Aldh2*^−/−^ knockout mice exhibit age-dependent deficits in motor performance responsiveness to levodopa and a significant loss of tyrosine hydroxylase (TH)-positive neurons in the substantia nigra (Wey et al., 2012). These data indicate that ALDH2 likely plays an important role in the pathogenesis of neurodegenerative diseases including PD.

In the present study, we investigated the protective effect of ALDH2 activation in rotenone-induced cellular and animal models for parkinsonism. Our data revealed that over-expression of ALDH2 enzyme confers a neuroprotective effect against rotenone-induced neuronal death and that ALDH2 small molecule activators, like Alda-1, may serve as a therapeutic agent for PD in the future.

## Materials and Methods

### Stable expression of wild-type (WT) ALDH2 or mutant ALDH2*2 in SH-SY5Y cells

Human neuroblastoma SH-SY5Y cells were grown in Dulbecco’s modified Eagle’s medium supplemented with Nutrient mixture F-12 (Ham) (1:1, v/v) (Gibco, Gaithersburg, MD, USA), 2 g/L sodium bicarbonate (Sigma-Aldrich), 100 units/ml penicillin, 100 μg/ml streptomycin and 10 % fetal bovine serum (FBS). Cells were grown at 37°C in a humidified air with 5% CO_2_. SH-SY5Y cells were grown to 80–90 % confluence and then sub-cultured into different culture plates. The cDNAs of WT ALDH2 (GenBank ID: BC002967) and (E504K) ALDH2*2 mutant were constructed and subcloned into a mammalian expression vector pcDNA3 (Invitrogen) containing FLAG-tag sequences (DYKDDDDK). Both plasmids were transfected to SH-SY5Y cells using Lipofectamine 2000 (Invitrogen). Two days after transfection, SH-SY5Y cells stably expressing WT ALDH2 and mutant ALDH2*2 were selected in the presence of 1.5 mg/ml G418 (geneticin sulfate) (Sigma-Aldrich). Positive clones were confirmed by Western blot analysis as described below and maintained in the medium containing 1 mg/ml G418.

### Preparation of primary culture of substantia nigra dopaminergic neurons

Primary cultured substantia nigra (SN) dopaminergic neurons were prepared as described previously (Wang et al., 2011). Briefly, substantia nigra was dissected from embryonic day 12 to 15 rats and cultured in DMEM/F12 medium containing pronase (0.5 mg/ml, Roche) and DNase I (0.3mg/ml, Roche) for 50 min at 37 °C. Tissue fragments were subsequently triturated, and dissociated cells were plated onto poly-L-lysine and collagen-coated six well dishes. SN neurons were cultured in DMEM/F12 medium supplemented with 5% fetal bovine serum, 5% horse serum and GDNF (glial cell line-derived neurotrophic growth factor; 25 ng/ml). 5′-fluoro-2′-deoxyuridine and uridine, which prevent proliferation of glial cells, were added into culture medium after 24 hours. Primary neuronal culture of substantia nigra consisted mainly of large oval-shaped dopaminergic neurons and small non-dopaminergic cells. In accordance with our previous studies (Wang et al., 2007, Wang et al., 2011), two subpopulations of neurons, large multipolar or oval-shaped tyrosine hydroxylase (TH)-positive dopaminergic cells (diameter = 25–30 μm), which was confirmed by TH immunostaining (data not shown), and small non-dopaminergic cells (diameter = 15–20 μm), were found in SN neuronal culture.

### Immunoblot analysis

Protein samples were prepared by homogenization of SH-SY5Y cells or SN neurons in SDS sample buffer. Subsequently, protein lysate or the immunocomplex was separated on 8 or 10% SDS-polyacrylamide gel and transferred to PVDF membrane. Then, the membrane was incubated at 4 °C overnight with one of the following diluted primary antibodies: (1) Anti-FLAG monoclonal antiserum (Sigma-Aldrich). (2) Polyclonal anti-Complex IV antibody (Sigma-Aldrich). (3) Polyclonal anti-cleaved active caspase 9 antibody (Cell Signaling Technology). (4) Polyclonal anti-cleaved active caspase 3 antiserum (Cell Signaling Technology). (5) Polyclonal anti-Bax antibody (Cell Signaling Technology). (6) Polyclonal anti-cytochrome c antiserum (Abcam). After the washes, the membranes were incubated with horse anti-mouse, anti-donkey or anti-rabbit horseradish peroxidase (HRP) linked secondary antibodies. Then, immunoreactive proteins were visualized by using an enhanced chemiluminescence kit (GE Biosciences). To confirm equal amount of protein sample loading, membranes were stripped and reblotted with monoclonal anti-actin antibody (Chemicon). Gel bands were quantified by a densitometer (Molecular Dynamics Model 375A) and normalized by reprobing the same blot for the actin signals.

### Subcellular expression of FLAG-tagged ALDH2

To analyze the subcellular distribution of ALDH2, mitochondrial and cytosolic fractions of SH-SY5Y cells expressing WT or (E504K) ALDH2*2 were prepared. Briefly, stable clones were lysed in ice-cold buffer containing 210 mM mannitol, 70 mM sucrose, 10 mM HEPES, pH7.3, 1 mM EGTA, 1 mM DTT, 5 μg/ml pepstain, 5 μg/ml leupetin, 5 μg/ml aprotinin, and 0.3 mM PMSF. Cells were homogenized using a Dounce tissue grinder on ice, and cell lysate was centrifuged at 500×g for 10 min at 4 °C. The supernatant was centrifuged at 9500×g for 9 min at 4 °C to pellet the mitochondrial fraction. The resulting supernatant was further centrifuged at 16,000×g for 20 min at 4 °C, and the final supernatant was used as cytosolic fraction. Protein samples (15 μg) of mitochondrial or cytosolic extracts were separated on a 10% SDS polyacrylamide gel and transferred to PVDF membrane. Then, FLAG tagged ALDH2 on the membrane was visualized by using a ECL protocol as described above. To verify the purity of mitochondrial fraction and detect possible mitochondrial contamination of cytosolic extract, membrane was stripped and reblotted with monoclonal anti-cytochrome c oxidase subunit IV (COX-IV), a mitochondrial marker (Molecular Probes). We verified that COX-IV is present in mitochondrial fraction and not detected in cytosolic extract.

### Measurement of ALDH2 activity

ALDH2 activity was determined in the samples, using aldehyde dehydrogenase 2 activity assay kit (Abcam), according to the manufacture’s recommendation. Briefly, the diluted proteins were added into the well of the microplate, coating specific anti-ALDH2 antibody, and enzymatic activity of ALDH2 was determined by monitoring the generation of NADH. The production of NADH was coupled to a reporter dye to yield a yellow colored reaction product whose concentration could be determined by measuring the increase in absorbance at 450 nm for 60 min on a Beckman DU800 spectrophotometer.

### Determination of 4-HNE level

4-HNE-histidine ELISA kit (Cell Biolabs) was used to measure the levels of 4-HNE in cellular or tissue extracts. Briefly, protein extracts or bovine serum albumin (BSA) standards were adsorbed onto a 96-wells microplate for 2 hours at 37°C. The anti-4-HNE-histidine antibody was used to probe with the 4-HNE-protein adduct content, which was subsequently measured by horseradish peroxidase (HRP)-linked secondary antibody and quantified by comparing with the standard curve of 4-HNE-BSA standards.

### Confocal imaging of mitochondrial membrane potential and reactive oxygen species (ROS) formation

For the determination of mitochondrial membrane potential (ΔΨm), SH-SY5Y cells or cultured SN dopaminergic neurons were loaded with a potential sensitive dye TMRM (tetramethylrhodamine methyl ester; 100 nM; Molecular Probes) in HEPES buffered saline (NaCl 140 mM, KCl 5 mM, MgCl_2_ 1 mM, CaCl_2_ 2 mM, glucose 10 mM, HEPES 5 mM, pH 7.3) for 10 min at room temperature. SH-SY5Y cells or SN neurons were then washed with HEPES buffered saline, and transferred to the recording chamber mounted on a Leica DM6000 microscope equipped with Leica TCS SP5 confocal spectral scanning system and a 100× oil immersion objective. TMRM was excited at 543 nm with a HeNe green laser, and the emitted fluorescent signal at 560–620 nm was collected. Z-stacks of 15 confocal TMRM fluorescence images (with an acquisition interval along the z-axis of 0.3 μm) were processed and analyzed by LAS AF software (Leica) as previously described (Wang et al., 2011).

Confocal MitoSOX Red staining was performed to visualize mitochondrial level of superoxide anion in SH-SY5Y cells and SN dopaminergic neurons. MitoSOX Red selectively targeted to the mitochondria is oxidized by superoxide and exhibits red fluorescence. SH-SY5Y cells and cultured SN neurons were incubated with 5 μM MitoSOX Red (Molecular Probes) for 10 min at 37 °C. After washout, MitoSOX Red was excited at 514 nm with an Ar-blue laser, and fluorescence signal was detected at 540–620 nm emission. MitoSOXRed fluorescence images were analyzed by Leica LAS AF software.

### Assays of cell survival

Cell survival was assessed by WST-8 [2-(2-methoxy-4-nitrophenyl)-3-(4-nitrophenyl)-5-(2,4-disulfophenyl)-2H-tetrazolium, monosodium salt] assays using Cell Counting Kit-8 (Fluka-Sigma-Aldrich, MO, USA). A total of 1×10^4^ SH-SY5Y cells were seeded per well in a 96-well plate. SH-SY5Y cells were administered with various concentrations of rotenone ranging from 0 to 500 ng/ml. After 24-hour incubation, 10 μl of WST-8 was added into each well in a humidified 5% CO_2_ atmosphere at 37°C for an additional 1 hour of incubation. The optical density (OD) was measured with a microplate reader (Bio-Rad, CA, USA) at 450 nm.

### Rotenone-induced mouse model of parkinsonism and Alda-1 treatment

C57BL/6 mice (2–3 months old) weighing 20–25 g were purchased from the National Laboratory Animal Center (Taipei, Taiwan). All animals used for this study were approved by the Institutional Animal Care and Use Committee of Chang Gung University. Mice were randomly divided into the following 6 groups: control group, Alda-1 treated group, rotenone treated group, Alda-1 plus rotenone treated group, MPTP (1-methyl-4-phenyl-1,2,3,6-tetrahydropyridine) treated group, and Alda-1 plus MPTP treated group. Mice were intraperitoneally (i.p.) injected with saline in the control group for 14 days. Mice treated with Alda-1 (50 mg/kg/day, i.p.) for 14 days served as the Alda-1 treated group. In rotenone treated group, mice were treated with rotenone (50 mg/kg/day, oral administration) for 14 days. For Alda-1 plus rotenone treated group, mice were intraperitoneally administered with Alda-1 (50 mg/kg) six hours before rotenone dosing. In addition, we tested the effect of Alda-1 on MPTP model for parkinsonism. In MPTP treated group, mice were injected with MPTP (40 mg/kg/day, i.p.) for 14 days. For Alda-1 plus MPTP treated group, mice were intraperitoneally administered with Alda-1 (50 mg/kg) six hours before MPTP dosing. Six animals from each group were used for the analysis of tyrosine hydroxylase (TH) immunohistochemistry.

### Quantification of SN tyrosine hydroxylase (TH)-positive neurons

For immunohistochemical analyses, mice were deeply anesthetized by chloral hydrate (450mg/kg, i.p.) and intracardially perfused with saline followed by 4% paraformaldehyde in 0.01 M phosphate-buffered saline (PBS), pH 7.4. The brains were rapidly removed from skull, postfixed overnight in 4% paraformaldehyde and stored in 30% sucrose at 4 °C. The brains were frozen and processed into 30 μm-thick coronal sections using a microtome cryostat (CM3000, Leica, Wetzlar, Germany) at −20 °C. The freeze-floating brain sections were rinsed in PBS and then incubated in 0.3% hydrogen peroxide for 15 min. The sections were washed in PBS containing 0.3% Triron X-100 (PBS-T) and then incubated with mouse monoclonal anti-tyrosine hydroxylase (TH) antibody (1:1000; Merck Millipore, Billerica, MA, USA) in PBS-T containing 10% normal goat serum overnight at 4 °C. After two washes with PBS-T, the sections were incubated with biotinylated secondary anti-mouse IgG antibody (Vector Laboratories, Burlingame, CA) in PBS-T for 1 hour at room temperature, followed by incubation in avidin-biotin complex (1:2000; Vector Laboratories, Burlingame, CA) in PBS-T for 1 hour at room temperature. Visualization of TH immunoreactivity was performed by incubating with 0.05% diaminobenzidine-HCl (DAB) for 2–5 min. TH-positive dopaminergic neurons were visualized and counted under a Leica DM2500 microscope equipped with an RET 2000R CCD camera (Qimaging, Surrey, BC, Canada) and a 3-axis computer-controlled MAC 600 motorized stage (Ludl Electronics, Hawthorne, NY, USA) with the aid of StereoInvestigator software (MBF Bioscience, Williston, VT, USA) as previously described (Chen et al., 2012). Each experimental analysis, 15–20 brain sections per mouse were processed.

### Chemicals

Rotenone (Sigma-Aldrich, MO, USA) was dissolved in dimethylsulfoxide (DMSO) (1 mM stock solution) and then diluted in culture medium. Alda-1 (N-(1,3-Benzodioxol-5-ylmethyl)-2,6-dichloro-benzamide) was provided by Dr. Daria Mochly-Rosen. All other chemicals used in this study are of the highest grade purchased from Sigma-Aldrich.

### Statistical analysis

All results are expressed as the mean ± SD value of n experiments. Statistical significance among multiple experimental groups was determined by one-way ANOVA followed by Dunnett’s test. Nonparametric Mann-Whitney U test (two-tailed) was used to determine the significant differences between two groups of data. Pearson correlation coefficient analysis was used to measure the correlation between two variables. A *P* value <0.05 was considered significant.

## Results

### Overexpression of wild-type human ALDH2, but not mutant human (E504K) ALDH2*2, protects against rotenone-induced cell death

**Figure 1:**
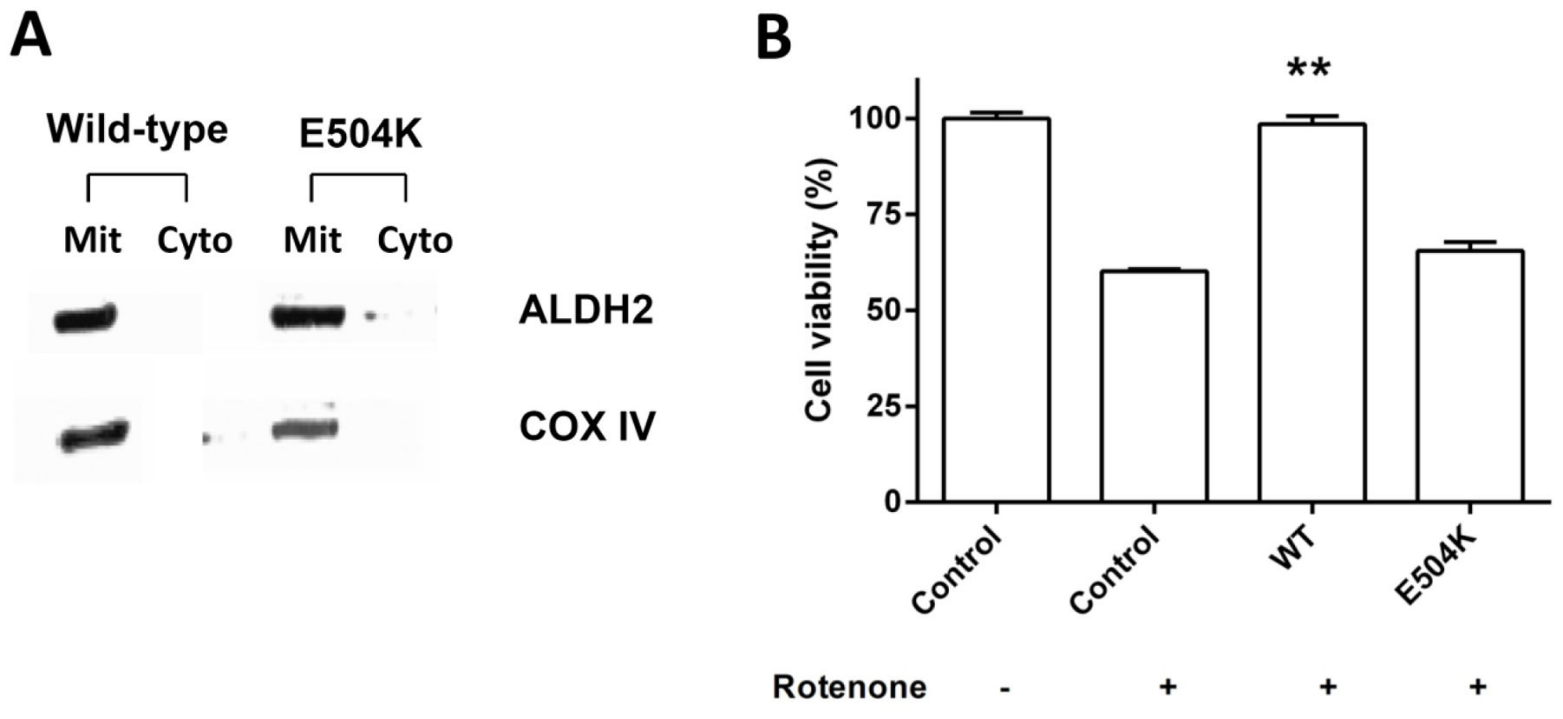
Overexpression of ALDH2 protects against rotenone-induced cell death. (A) SH-SY5Y cells were stably transfected with cDNA of FLAG-tagged wild-type (WT) or inactive (E504K) ALDH2*2. Stable clones were subfractionated into mitochondrial (Mit) and cytosolic (Cyto) fractions. Western blot analysis using anti-FLAG antibody indicated that ALDH2 was selectively expressed in the mitochondrial fraction of SH-SY5Y cells. Cytochrome c oxidase subunit IV (COX-IV) was used as an internal control for mitochondrial fraction. (B) Control cells and stable clones expressing WT ALDH2 or (E504K) ALDH2*2 were plated onto 96-well plates and allowed to adhere overnight. Cell Counting Kit-8 was used to analyze cell survival. Rotenone treatment (100 nM) for 24 hours caused a significant reduction of cell viability in control cells. Overexpression of WT ALDH2, but not (E504K) ALDH2*2 significantly protected against rotenone-induced cell death. Each bar represents the mean ± SD value of five independent experiments. *p< 0.05, **p<0.01, ***p<0.001 compared to rotenone-treated cells. Mit: mitochondrial; Cyto: cytosol fraction; WT: wild-type; E504K: (E504K) ALDH2 mutant.

To examine the potential neuroprotective property of ALDH2, SH-SY5Y cells stably expressing FLAG-tagged wild-type (WT) ALDH2 or (E504K) mutant ALDH2*2 were established. The Glu504Lys (E504K) polymorphism in the ALDH2, which exists in 35–57% of East Asians (Li et al., 2009), has reduced ALDH2 activity (Chen et al., 2008). The control stable cells, transfected with an empty pcDNA3-FLAG plasmid vector, were also established. Subcellular distribution of ALDH2 protein was analyzed, and Western blot analysis using anti-FLAG antibody showed that WT or mutant (E504K) ALDH2 were selectively expressed in the mitochondrial fraction of stable clones (Fig. 1A). Overexpression of WT ALDH2, but not E504K ALDH2, significantly elevated ALDH2 activity compared to control stable cells (2.27 fold, *p*=0.0001 ) (Supplementary figure 1). Rotenone, a natural occurring pesticide, is a well-known inhibitor of mitochondrial complex I. Many studies have demonstrated that rotenone selectively induces degeneration of SN dopaminergic neurons and neuropathological features of PD (Alam and Schmidt, 2002). To evaluate the neuroprotective effect of ALDH2 overexpression, stable clones expressing WT ALDH2 or (E504K) ALDH2*2 were treated with 100 nM rotenone for 24 hours. Cell survival was assayed by Cell Counting Kit-8. Rotenone treatment decreased cell viability of control cells (60.1±0.6% of control) (Fig. 1B). Compared to rotenone-treated control cells, expression of WT ALDH2 significantly attenuated rotenone-induced cell death (98.5±2.1% of control, *p*=0.008), whereas overexpression of (E504K) ALDH2*2 was ineffective in preventing rotenone-induced cytotoxicity (Fig. 1B).

### Expression of WT ALDH2 prevents rotenone-induced loss of mitochondrial membrane potential (ΔΨm) and generation of reactive oxygen species (ROS)

**Figure 2:**
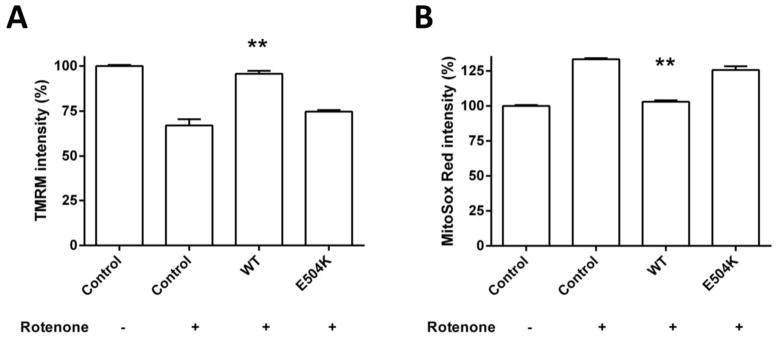
Overexpression of ALDH2 blocks rotenone-induced loss of mitochondrial membrane potential and generation of reactive oxygen species. (A) Confocal TMRM staining was performed to quantify hyperpolarized mitochondrial membrane potential (ΔΨm). TMRM fluorescence intensity was significantly decreased in rotenone-treated cells. Note that overexpression of WT ALDH2 prevented rotenone-induced reduction in TMRM fluorescence signal (95.7±1.6% v.s. 67±3.5%, p=0.002). (E504K) ALDH2*2 mutant failed to restore TMRM fluorescence intensity. (B) Confocal imaging of MitoSox Red was performed to evaluate mitochondrial ROS production. MitoSox Red fluorescence signal was significantly increased in rotenone-treated cells. Overexpression of WT ALDH2 significantly prevented rotenone-induced increase in MitoSox Red fluorescence intensity (103.1±1% v.s. 133.4±0.8%, *p*=0.002). Overexpression of (E504K) ALDH2*2 was ineffective in inhibiting rotenone-induced increase in MitoSox Red signal. Each bar shows the mean ± SD value of 25–35 cells. *p< 0.05, **p<0.01, ***p<0.001 compared to rotenone-treated cells.

Since rotenone causes cell death by inducing mitochondrial dysfunction, we sought to determine whether the cytoprotective effect of ALDH2 is mediated through regulation of the mitochondrial ΔΨm and mitochondrial ROS generation. Confocal image of TMRM fluorescence was performed to visualize hyperpolarized ΔΨm of stable clones expressing WT ALDH2 or (E504K) ALDH2*2. The selective accumulation of cationic dye TMRM in the mitochondria of healthy cells is driven by hyperpolarized ΔΨm. Therefore, TMRM fluorescence intensity is an indicator of hyperpolarized ΔΨm. Compared to control cells, TMRM fluorescence signal was significantly reduced in rotenone (100 nM)-treated cells (67±3.5% of control) (Fig. 2A). Overexpression of WT ALDH2 attenuated rotenone-induce decrease in TMRM fluorescence intensity as compared with rotenone-treated control cells (95.7±1.6% v.s. 67±3.5%, *p*=0.002) (Fig. 2A). In contrast to WT ALDH2, (E504K) ALDH2*2 was ineffective in preventing rotenone-induced loss of ΔΨm (Fig. 2A).

Confocal MitoSox Red imaging was performed to visualize mitochondrial level of superoxide anion, a major ROS produced by the mitochondria. MitoSox Red is selectively targeted to the mitochondria, and exhibits red fluorescence as a result of oxidation by superoxide anion. In comparison with control cells, MitoSox Red fluorescence intensity was increased in rotenone (100 nM)-treated cells (133.4±0.8% of control) (Fig. 2B). Overexpression of WT ALDH2 prevented rotenone-induced increase in MitoSox Red fluorescence signal (103.1±1% v.s. 133.4±0.8%, *p*=0.002), while (E504K) ALDH2*2 was ineffective in decreasing rotenone-induced ROS production (Fig. 2B). Overexpression of WT ALDH2 alone did not affect the intensity of TMRM and MitoSox Red fluorescence (data not shown).

### The ALDH2 activator, Alda-1, prevents rotenone-induced loss of mitochondrial membrane potential and ROS generation in a dose-dependent manner

**Figure 3:**
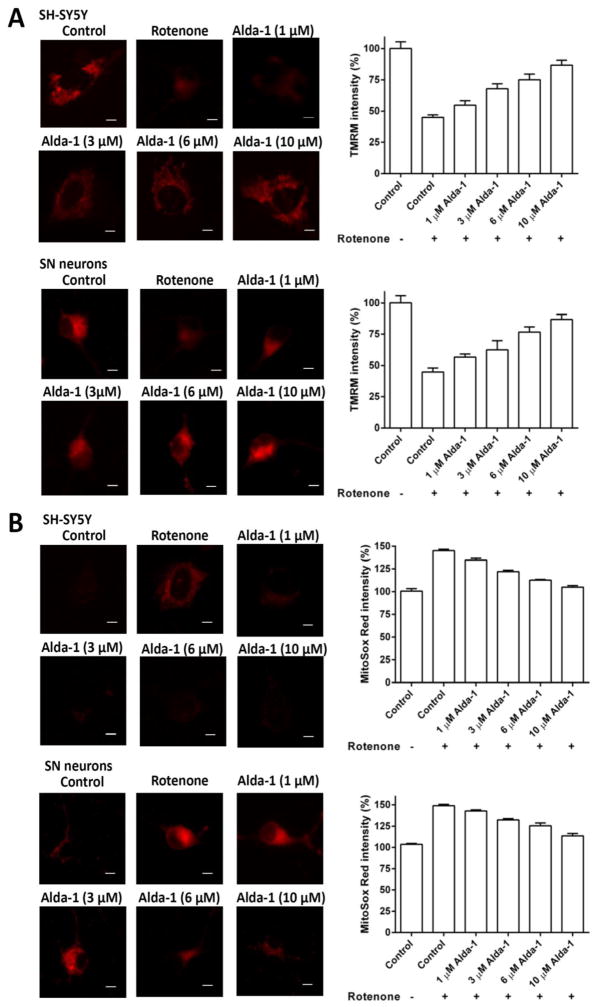
ALDH2 activation by Alda-1 prevents rotenone-induced loss of mitochondrial membrane potential and ROS production in a dose-dependent manner. (A) SH-SY5Y cells and SN dopaminergic neurons were pretreated with various concentrations of Alda-1 (1–10 μM) for 24 hours and then treated with 100 nM rotenone for additional 24 hours. Compared to rotenone-treated cells, Alda-1 administration attenuated rotenone-induced reduction in TMRM fluorescence intensity of SH-SY5Y cells (r=0.942, *p*<0.01) and SN dopaminergic neurons (r=0.941, *p*<0.01) in a dose-dependent manner. (B) In contrast to rotenone-treated cells, Alda-1 treatment blocked rotenone-induced increase in MitoSox Red signal of SH-SY5Y cells (r=0.923, *p*<0.01) and SN dopaminergic neurons (r=0.916, *p*<0.01) in a concentration-dependent manner. Scale bar is 10 μm. Each bar represents the mean ± SD value of 25–35 cells or neurons. *p< 0.05, **p<0.01, ***p<0.001 compared to rotenone-treated cells.

Alda-1 (N-(1,3-Benzodioxol-5-ylmethyl)-2,6-dichloro-benzamide) is a potent activator of ALDH2 (Chen et al., 2008). Compared to control cells, treatment of Alda-1 significantly increased ALDH2 activity in SH-SY5Y cells (1.92 fold, *p*<0.0001) (Supplemental figure 1). To determine whether Alda-1 could ameliorate rotenone-induced mitochondrial dysfunction, SH-SY5Y cells and cultured SN dopaminergic neurons confirmed by TH-immunostaining (data not shown) were pretreated with various concentrations of Alda-1 (1–10 μM) for 24 hours and then treated with 100 nM rotenone for additional 24 hours. Similar to the observations described above, rotenone treatment caused a decrease of ΔΨm and increased ROS generation in SH-SY5Y cells and cultured SN dopaminergic neurons. Alda-1 treatment significantly ameliorated rotenone-induced decrease of ΔΨm in SH-SY5Y cells (Pearson Correlation Coefficient, r=0.942, *p*<0.01) and SN dopaminergic neurons (r=0.941, *p*<0.01) in a concentration-dependent manner (Fig. 3A). In contrast to rotenone-treated cells, Alda-1 treatment blocked rotenone-induced increase in MitoSox Red signal of SH-SY5Y cells (r=0.923, *p*<0.01) and SN dopaminergic neurons (r=0.916, *p*<0.01) in a concentration-dependent manner (Fig. 3B). Administration of 10 μM Alda-1 alone did not affect signals of TMRM and MitoSox Red fluorescence in the absence of rotenone (data not shown).

### Activation of ALDH2 suppresses rotenone-induced increase of cytosolic Bax, cytochrome c, active caspase-9 and active caspase-3

**Figure 4:**
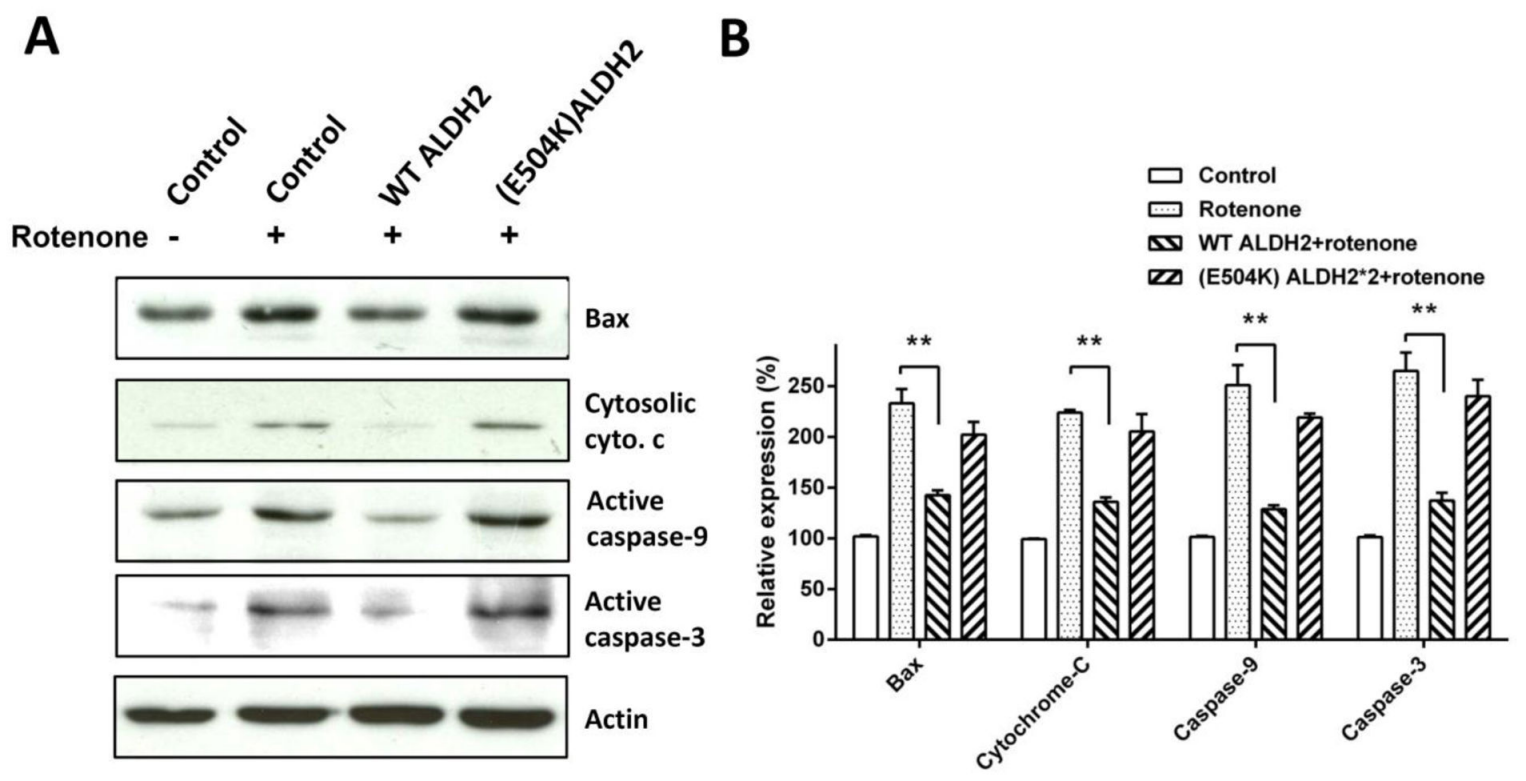
Overexpression of WT ALDH2 blocks rotenone-induced upregulation of cytosolic Bax, cytochrome c, active caspase-9 and active caspase-3 protein levels. (A) Treatment with rotenone significantly increased protein levels of cytosolic Bax, cytochrome c, active cleaved caspase-9 and active cleaved caspase-3 in the cytosol fraction of control SH-SY5Y cells. Compared to rotenone-treated control cells, overexpression of WT ALDH2 ameliorated rotenone-induced increase in cytosolic protein levels of Bax, cytochrome c, active caspase-9 and active caspase-3. Expression of (E504K) ALDH2 failed to inhibit rotenone-induced increased protein levels of cytosolic Bax, cytochrome c, active caspase-9 and active caspase-3. (B) Level of apoptosis related proteins was quantified by densitometer. Each bar shows the mean ± SD value of six independent experiments. *p< 0.05, **p<0.01, ***p<0.001 compared to rotenone-treated cells.

Activation of mitochondrial apoptotic pathway leads to neuronal death observed in several neurodegenerative diseases including Parkinson’s disease. Previous studies reported that rotenone exposure induces apoptotic cell death via the release of cytochrome c from mitochondria by Bax and subsequent activation of caspases, including caspase-9 and caspase-3 (Jayaraj et al., 2013, Tamilselvam et al., 2013, Wang et al., 2014). As expected, in control SH-SY5Y cells treated with 100 nM rotenone, Western blot analysis showed increase in cytosolic levels of Bax, cytochrome c, active caspase-9 and active caspase-3 (Fig. 4). Compared to rotenone-treated control cells, overexpression of WT ALDH2 significantly blocked rotenone-induced Bax expression, release of cytochrome c, activation of caspase-9 and caspase-3 (Fig. 4B). In contrast, expression of (E504K) ALDH2*2 did not attenuate rotenone-induced upregulation of protein expression of cytosolic Bax, cytochrome c, active caspase-9 and active caspase-3.

**Figure 5:**
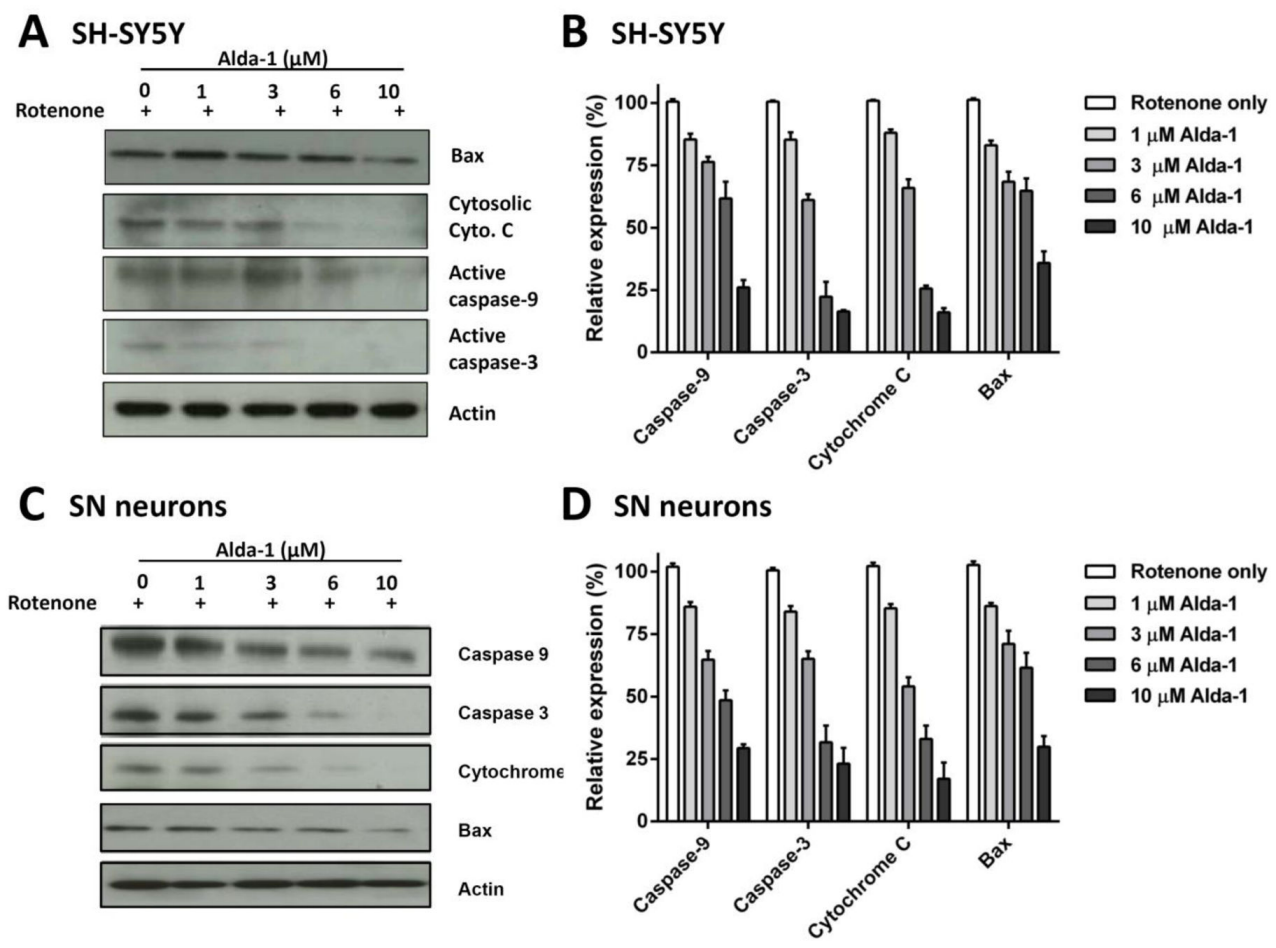
Alda-1 attenuates rotenone-induced activation of mitochondrial apoptotic pathway. (A, C) Compared to rotenone-treated cells, Alda-1 administration greatly attenuated rotenone-induced increase in protein level of cytosolic Bax, cytochrome c, active caspase-9 and active caspase-3 in SH-SY5Y cells and cultured SN neurons. (B, D) Quantification of level of apoptosis related proteins measured by the use of a densitometer showed that Alda-1 treatment inhibited rotenone-induced increase in cytosolic Bax, cytochrome c, active caspase-9 and active caspase-3 in SH-SY5Y cells and cultured SN neurons in a concentration-dependent manner. Each bar represents the mean ± SD value of at least three independent experiments.

Next, we examined activation of mitochondrial-dependent apoptotic pathway induced by rotenone toxicity in SH-SY5Y cells and cultured SN neurons pretreated in the presence of various concentrations of Alda-1 (1–10 μM) for 24 hours. In agreement with the result of ALDH2 overexpression experiment above, Western blot analyses demonstrated that Alda-1 treatment inhibited rotenone-induced increase in cytosolic Bax, cytochrome c, active caspase-9 and active caspase-3 in SH-SY5Y cells (Fig. 5A & 5B) and cultured SN neurons (Fig. 5C & 5D), in a dose-dependent manner.

### Alda-1 ameliorates rotenone-induced death of SN TH^+^ dopaminergic neurons *in vivo*

**Figure 6:**
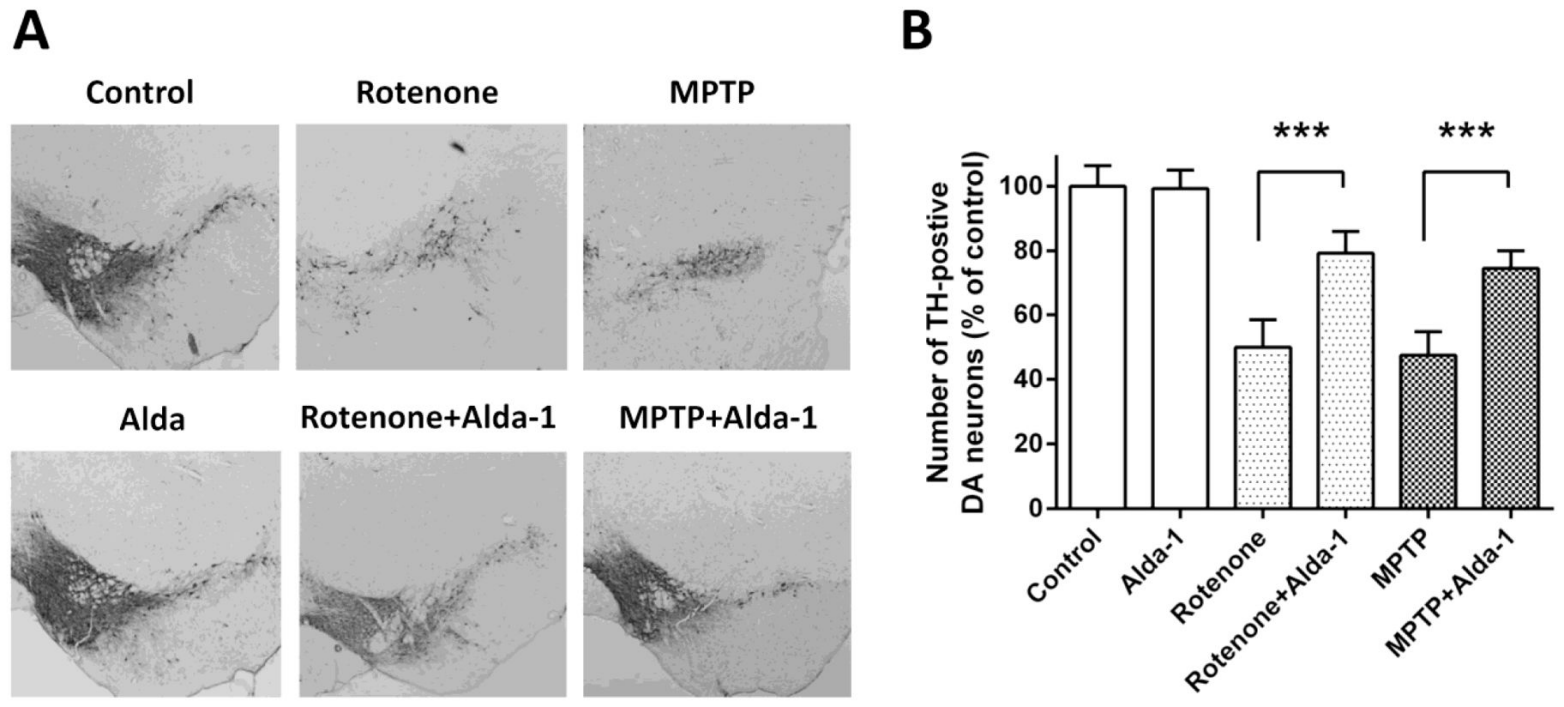
ALDH2 activator, Alda-1, exerts a neuroprotective effect against rotenone-induced death of SN TH^+^ dopaminergic neurons. (A) Immunohistochemical staining demonstrated the loss of TH^+^ dopaminergic neurons in the SN after rotenone administration. Alda-1 treatment ameliorated rotenone- or MPTP-induced reduction in the number of SN TH^+^ dopaminergic neurons. (B) Quantification of SN TH^+^ dopaminergic neurons showed that Alda-1 reduced rotenone- or MPTP-induced loss of SN dopaminergic neurons. Administration of Alda-1 greatly reduced rotenone-induced death of SN TH^+^ dopaminergic neurons. Alda-1 treatment was also effective to significantly reduce MPTP-induced loss of SN TH^+^ dopaminergic neurons. Each bar shows the mean ± SD value from 6 animals. ***p<0.001 compared to rotenone- or MPTP-treated mice.

Recent studies have suggested that rotenone-induced mouse model of parkinsonism proved to be a valuable tool in mimicking the pathological features of PD and used for the development of new neuroprotective strategies (Pan-Montojo et al., 2012, Parameshwaran et al., 2012, Shinomol et al., 2012, Yoshimoto et al., 2012, Chandran and Muralidhara, 2013, Weetman et al., 2013, Gokul and Muralidhara, 2014). To examine neuroprotective effect of Alda-1 in a rotenone-induced mouse model of parkinsonism, immunohistochemical staining of tyrosine hydroxylase (TH) was conducted to visualize degeneration of SN dopaminergic neurons. Mice were randomly divided into the following 6 groups: (1) control group (saline i.p. for 14 days); (2) Alda-1 treated group (50 mg/kg/day, i.p. for 14 days); (3) rotenone treated group (50 mg/kg/day, oral administration for 14 days); (4) Alda-1 plus rotenone treated group (Alda-1 50 mg/kg/day six hours before rotenone 50mg/kg/day for 14 days); (5) MPTP treated group (40 mg/kg/day, i.p. for 14 days); and (6) Alda-1 plus MPTP treated group (Alda-1 50 mg/kg/day 6 hours before MPTP 40 mg/kg/day for 14 days). Each group contained 6 mice. In comparison with control group mice, administration of either rotenone (49.9±8.5% of control, *p*<0.001) or MPTP (47.5±7.4% of control, *p*<0.001) caused a marked reduction in number of TH^+^ dopaminergic neurons in the SN (Fig. 6). In contrast, Alda-1 treatment greatly reduced rotenone-induced loss of SN TH^+^ dopaminergic neurons (76.3±5.7% of control v.s. 49.9±8.5% of control, *p*<0.001) (Fig. 6). Alda-1 treatment was also effective to reduce MPTP-induced death of SN TH^+^ dopaminergic neurons (74.3±5.7% of control v.s. 47.5±7.4% of control, *p*<0.001) (Fig. 6). Alda-1 alone did not affect the number of SN TH^+^ dopaminergic neurons.

### ALDH2 activation prevents 4-HNE accumulation in rotenone- or MPTP-induced cellular or mouse model of parkinsonism

ALDH2 is a key enzyme in detoxifying aldehydes, such as 4-HNE, in the brain. Thus, we studied whether the activation of ALDH2 could ameliorate rotenone- or MPTP-induced elevation of 4-HNE *in vitro* and *in vivo*. Rotenone (100 nM) treatment caused a significantly increase of 4-HNE level in SH-SY5Y control cells. Overexpression of WT ALDH2, but not E504K ALDH2, significantly prevented rotenone (100 nM)-induced accumulation of 4-HNE compared to rotenone-treated cells (Supplementary figure 2A). Administration of Alda-1 (1–10 μM) significantly ameliorated rotenone-induced increase of 4-HNE in SH-SY5Y cells (r=0.982, *p*<0.01) and cultured SN dopaminergic neurons (r=0.969, *p*<0.01) in a concentration-dependent manner (Supplementary figure 2B and 2C). In the rotenone (50 mg/kg/day, oral administration for 14 days)- or MPTP (40 mg/kg/day, i.p. for 14 days)-induced mouse model of parkinsonism, Alda-1 treatment (50 mg/kg/day, i.p.) significantly reduced rotenone- or MPTP-induced accumulation of 4-HNE in the SN (Supplementary figure 2D).

## Discussion

This study shows that increased ALDH2 activity by either genetic overexpression or pharmacological activation is effective to protect against rotenone-induced cell death. The neuroprotection results from decreased ROS accumulation, decreased depolarization of mitochondrial membrane potential and inhibition of mitochondrial apoptotic pathway activation. These results indicate that ALDH2 plays an important role on maintaining normal mitochondrial function and that ALDH2 activation is effective in ameliorating mitochondrial dysfunction and inhibiting mitochondria-mediated apoptosis caused by neurotoxin.

Multiple lines of evidence suggest an important role of oxidative damage and mitochondrial dysfunction in the pathogenesis of PD (Schapira, 2008, Perier and Vila, 2012, Dexter and Jenner, 2013). The mitochondria are both a source and a target of toxic ROS and oxidative stress. Mitochondrial dysfunction can lead to cell cells by the accumulation of oxidized products such as aldehydes and isoprostanes from lipid peroxidation, protein carbonyls from protein oxidation, and base adducts from DNA oxidation. A direct relation between mitochondrial damage and cell death is supported by the observation of a consistent deficit in the subunits and activity of mitochondrial complex I of the electron transport chain in blood platelets and SNpc of PD patients (Schapira, 2008). Many studies have shown that exposure to certain pesticides such as rotenone or paraquat, industrial wastes, and environmental toxin (most of which are toxic to the mitochondria) is associated with PD (Gorell et al., 1998, Betarbet et al., 2000, Wirdefeldt et al., 2011, Kieburtz and Wunderle, 2013). Moreover, nigrostriatal DA neurons in general are under tremendous oxidative stress due to redox cycling of catechols to reactive aldehydes such as DOPAL, MDA and 4-HNE, leading to increased generation of detrimental ROS (Ohta et al., 2004, Marchitti et al., 2007, Panneton et al., 2010, Goldstein et al., 2013). Several genes associated with PD, including α-synuclein, parkin, DJ-1, PINK1 and LRRK2, are also linked to oxidative damage and mitochondrial dysfunction-related to the pathogenic mechanism of PD (Bogaerts et al., 2008, Schapira, 2008). Therefore, protection against the oxidative damage and mitochondrial dysfunction has a great potential as a target for PD therapy.

The production of reactive aldehydes and impaired aldehyde detoxification capacity likely participate in the pathogenesis of PD (Ohta et al., 2004, Marchitti et al., 2007, Chen et al., 2014). ALDH2 is a critical enzyme for the metabolism of ethanol-derived acetaldehyde and plays a key role in oxidizing endogenous aldehydic products that arise from lipid peroxidation under oxidative stress, including 4-HNE, MDA, and DOPAL (Chen et al., 2014). 4-HNE induces mitochondria-mediated apoptosis in PC12 cells (Siddiqui et al., 2012) and inhibits dopamine transport in rat striatal synaptosomes (Morel et al., 1998). Increased immunoreactivity of 4-HNE has been found in the Lewy bodies of PD patients (Castellani et al., 2002). Injections of DOPAL selectively kills dopaminergic neurons (Burke et al., 2003) and triggers a behavioral phenotype (rotational asymmetry) consistent with other PD animal models (Panneton et al., 2010). Moreover, overexpression of ALDH2 attenuates neurotoxicity induced by 4-HNE in cultured primary hippocampal neurons (Bai and Mei, 2011). Mice null for Aldh1a1 and Aldh2 exhibit age-dependent deficits in motor performance in response to L-DOPA; these mice also have a significant loss of neurons immunoreactive for tyrosine hydroxylase (TH) in the substantia nigra as well as reduction of dopamine and metabolites in the striatum (Wey et al., 2012). Inhibition of ALDH by pesticides leads to accumulation of reactive dopamine metabolite DOPAL, preferential degeneration of dopaminergic neurons and the development of PD (Fitzmaurice et al., 2013). Together, these studies support ALDH2 as an important link for the etiology of PD.

Recent studies in humans demonstrated that exposures to ALDH-inhibiting pesticides were associated with 2- to 6-fold increases in PD risk and, importantly, the genetic variant of ALDH2*2 exacerbated PD risk in subjects exposed to ALDH-inhibiting pesticides (Fitzmaurice et al., 2013, Fitzmaurice et al., 2014). These findings suggest that gene-environment interaction plays an important role in the pathogenesis of PD. Consistent with this observation, we show here that overexpression of wild-type ALDH2 protects against rotenone (a common pesticide)-induced cell death.

In the present study, our results showed that intraperitoneal administration of Alda-1, a potent activator of ALDH2 (Chen et al., 2008), significantly prevented rotenone- or MPTP-induced loss of SN TH^+^ dopaminergic neurons *in vivo*. A recent study reported that Alda-1 also increased the activity of ALDH1A1 (Kotraiah et al., 2013). Overexpression of ALDH1A1 has been shown to reduce oxidation-induced cytotoxicity in SH-SY5Y cells (Zhang et al., 2010). Therefore, it is possible that in addition to activating ALDH2, Alda-1 also exert its neuroprotective effect against rotenone- or MPTP-induced neurotoxicity by activating ALDH1A1.

Parkinson’s disease is a progressive neurodegenerative disorder. Current treatment for PD is mainly symptomatic. There is therefore a great medical need to develop disease-modifying treatments that could halt or delay disease progression in PD (Chen et al., 2014). In this context, it is interesting to note that inhibitors of monoamine oxidase (MAO) have been shown to slow down PD progression early in the disease (Olanow et al., 2009, Dexter and Jenner, 2013). The discovery of potent aldehyde dehydrogenase activator compounds, such as Alda-1, holds a great potential for clinical application for PD to be used alone or together with other therapies, such as MAO inhibitors.

In summary, the present study showed that overexpression of wild-type ALDH2 or application of an ALDH2 small molecule activator, Alda-1, protects against rotenone-induced cell death in SH-SY5Y cells or cultured SN neurons by preventing rotenone-induced depolarization of mitochondrial membrane potential, ROS production, and activation of mitochondrial apoptotic pathway. In rotenone-induced mouse model of parkinsonism, ALDH2 activation using Alda-1 also attenuates rotenone-induced loss of SN TH^+^ dopaminergic neurons in mice. These results suggest that ALDH2 activation has a neuroprotective role in PD and that an ALDH2 activator, such as Alda-1, may serve as a neuroprotective agent for PD therapy.

## Supplementary Material

1

2

## Highlights

- Overexpression of wild-type ALDH2 reduces rotenone-induced cell death.
- Alda-1 is effective in protecting against rotenone-induced apoptotic cell death.
- Alda-1 prevents rotenone-induced ROS accumulation and depolarization of ΔΨm.
- Alda-1 significantly ameliorates rotenone or MPTP-induced cell death in the SN.
