# Supplementary figures and images for: Neuroprotective effects of aldehyde dehydrogenase 2 activation in rotenone-induced cellular and animal models of parkinsonism
Source: Exp Neurol. Author manuscript; Available in PMC 2016 Jan 1. (PMC4415848; doi:10.1016/j.expneurol.2014.09.016)

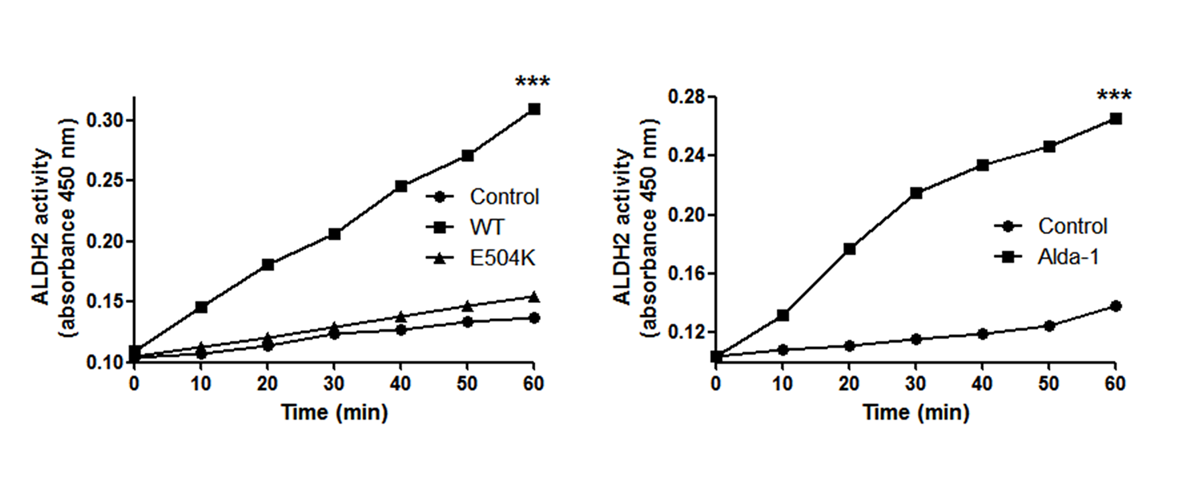

Supplement: 1 [file NIHMS660840-supplement-1.tif]

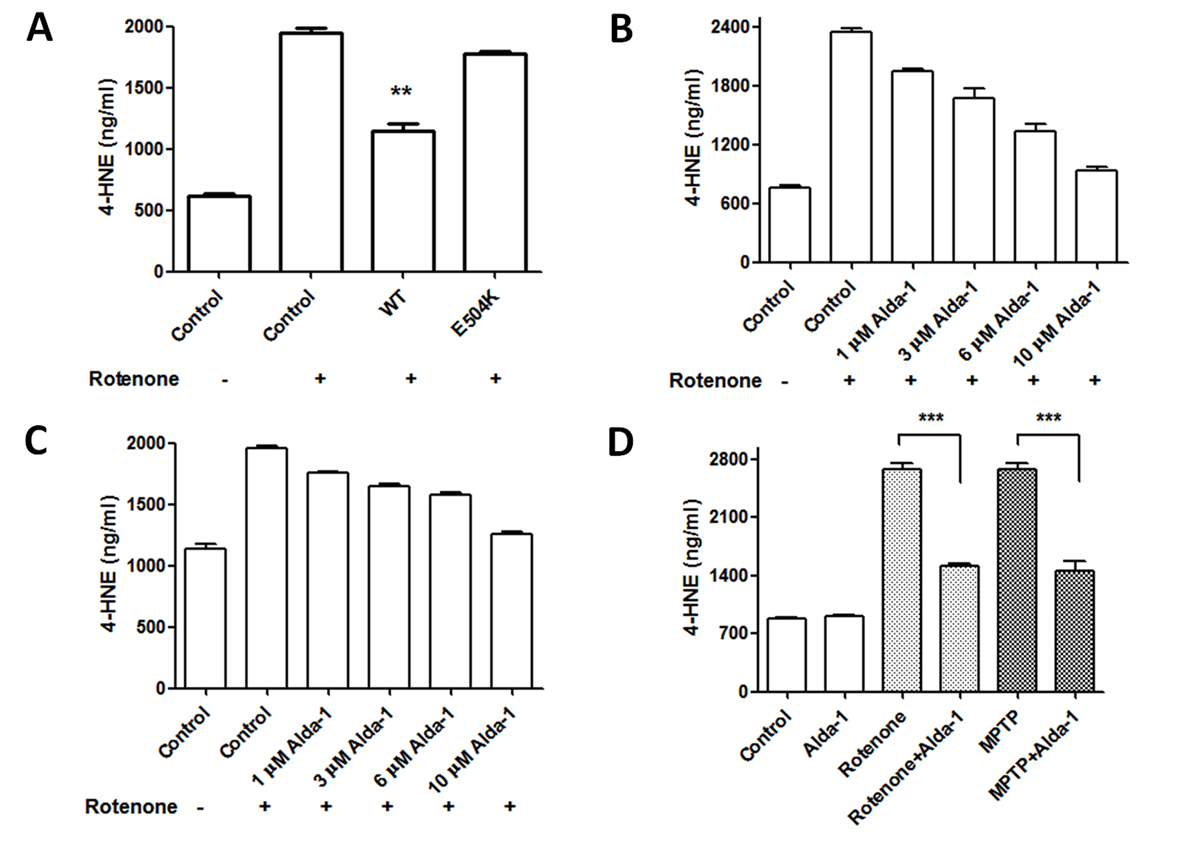

Supplement: 2 [file NIHMS660840-supplement-2.tif]
